# 

**Published:** 1856-03

**Authors:** 


*To Correspondents.*

Communications have been received from Dr. G. W. Adams, of Clinton, Ill.; Dr. S. O. Long, of Big Rock, Ill.; Dr. C. A. Hathwell, Virginia, Ill.; Dr. L. C. Lane, of Henderson, Ill.; Dr. D. W. Stormont, of Illinois; Dr. G. W. Hall, of Bainbridge, Ind.; and Dr. J. S. Collings, of Boxley, Ind. Letters merely remitting money, are sufficiently acknowledged in the list of receipts on the second page of the cover. Dr. H. G. Dunbar writes us some good advice, and among other things, complains of the *non*-reception of the January number of the Journal. His letter is dated "Beardstown, Feb. 25," and postmarked "Lancaster, Feb. 28." We have examined our mail list for each of the above places, but find the name of no such subscriber. At what post-office does Dr. Dunbar receive his Journal?

---

*Notice to former Subscribers.*

Some months since, notice was given that the names of all subscribers on our mail list, who were indebted for the Journal more than *one* year, would be stricken off on the first of Feb., 1856. In accordance with this notice, we have crossed off 215 names, not one of which has had any credit on the books during the last *four years.* They consequently stand indebted for volumes 1, 2, 3, and 4. As *three* of these volumes belong to the former Publisher, Mr. J. F. Balantyne, he has taken the accounts into his own hands, and will endeavor to collect them as he sees proper. All letters, however, in relation to the business of the Journal since January, 1855, should be addressed exclusively to N. S. Davis, Chicago, Illinois.

---

*New Medical Journal.*

We have received the first number of a new Journal, published at Detroit, Michigan, under the title of "The Medical Independent and Monthly Review of Medicine and Surgery." It is edited by Henry Goadley, M.D., F.L.S., &c. &c.; Edward Kane, M.D. and Prof.; L. G. Robinson, M.D. Published monthly by S. D. Elwood & Co., Detroit. Terms, $2,00 per annum.

### RECEIPTS ON SUBSCRIPTIONS,

FROM FEB. 15TH, TO MARCH 15TH, 1856.

**ILLINOIS.**

| | | |
|---|---|---|
| Dr. J. B. Samuel, | Vol. 4, | $2 00 |
| " J. B. Glass, | Vol. 4 & 5, | 4 00 |
| " W. S. Van Cleve, | Vol. 4, | 2 00 |
| " G. W. Kittell, | " 5, | 2 00 |
| " J. Perry, | " 5, | 2 00 |
| " C. A. Hathwell, | " 4, | 2 00 |
| " S. M. Meeker, | Vol. 4 & 5, | 4 00 |
| " A. G. Randall, | Vol. 5, | 2 00 |
| " R. S. Hitt, | " 5, | 2 00 |
| " H. W. Kreider, | " 5, | 2 00 |
| " J. Keenon, | " 5, | 2 00 |
| " H. C. Donaldson, | Vol. 4 & 5, | 4 00 |
| " C. Secrest, | Vol. 5, | 2 00 |
| " E. Farmer, | " 4, | 2 00 |
| " Z. H. Madden, | Vol. 4 & 5, | 4 00 |
| " J. W. Trabue, | Vol. 5, | 2 00 |
| " C. D. Henton, | " 5, | 2 00 |
| " J. B. E. Albright, | Vol. 4 & 5, | 4 00 |
| " W. C. Morris, | Vol. 5, | 2 00 |
| " J. M. Williamson, | " 5, | 2 00 |
| " J. Henderson, | " 5, | 2 00 |
| " Abner Hard, | " 4, | 2 00 |
| " Alex. Taylor, | " 5, | 2 00 |
| " V. C. McClure, | " 4, | 2 00 |
| " Bailey Ragon, | " 5, | 2 00 |
| " J. H. Robinson, | Vol. 4 & 5, | 3 00 |
| " B. E. Dodson, | Vol. 4, | 2 00 |
| " J. C. Carben, | Vol. 3, 4 & 5, | 6 00 |
| " E. S. Morey, | Vol. 5, | 2 00 |
| " C. M. Daniels, | " 4, | 2 00 |
| " J. W. Spaulding, | " 4, | 2 00 |
| " John Walker, | " 5, | 2 00 |
| " P. Kirwan, | " 4, | 2 00 |
| " C. Hard, | " 4, | 2 00 |
| " R. M. McArthur, | " 4, | 2 00 |
| " D. D. Thompson, | " 5, | 2 00 |
| " H. W. Hopkins, | " 5, | 2 00 |
| " Geo. T. Allen, | Vol. 4 & 5, | 4 00 |
| " A. M. D. Hughes, | Vol. 5, | 2 00 |
| " John Gregory, | " 5, | 2 00 |
| " E. H. Patton, | " 4, | 2 00 |
| " Thos. Hall, | Vol. 4 & 5, | 4 00 |
| " John B. Hunt, | Vol. 5, | 2 00 |
| " N. P. Golliday, | " 4, | 2 00 |

**INDIANA.**

| | | |
|---|---|---|
| Dr. Samuel Kelly, | Vol. 3 & 4, | $5 00 |
| " O. H. Martin, | " 4 & 5, | 5 00 |
| " J. W. Woodard, | Vol. 4, | 2 00 |
| " W. F. Green, | " 5, | 2 00 |
| " J. W. Green, | " 5, | 2 00 |
| " Latta & Jackson, | " 4, | 2 00 |
| " J. A. Adrain, | " 5, | 2 00 |
| " C. West, | Vol. 3 & 4, | 4 00 |
| " H. G. Chrestien, | Vol. 5, | 2 00 |
| " J. W. Perry, | " 4, | 2 00 |
| " S. B. Harriman, | " 4, | 2 00 |
| " Henry Wehman, | " 5, | 2 00 |
| " R. Winton, | " 5, | 2 00 |
| " A. J. Mirrick, | " 4, | 2 00 |
| " M. H. Bonner, | " 5, | 2 00 |
| " R. A. Perkins, | " 4, | 2 00 |
| " A. M. Dewey, | " 5, | 2 00 |
| " G. B. Grubb, | " 5, | 2 00 |

**IOWA.**

| | | |
|---|---|---|
| Dr. A. B. Ireland, | Vol. 4, | 2 00 |
| " H. C. Rawson, | " 5, | 1 00 |
| " L. Carpenter & Son, | " 5, | 2 00 |

**WISCONSIN.**

| | | |
|---|---|---|
| Dr. Hugh Russell, | Vol. 4, | 2 00 |
| " H. L. Coon, | " 4, | 2 00 |
| " Hanson Hurd, | Vol. 1, 2, 3, 4, | 8 00 |
| " Roswell Eaton, | Vol. 5, | 2 00 |
| " Benj. Durham, Jr. | " 5, | 2 00 |
| " J. P. Hamilton, | " 4, | 2 00 |
| " J. H. Sudduth, | " 5, | 2 00 |
| " J. B. Moffatt, | " 4, | 2 00 |
| " L. B. W. Johnson, | " 4, | 2 00 |
| " J. Rogers, | " 5, | 2 00 |
| " D. LaCount, | " 4, | 2 00 |
| " C. Nettleton, | " 5, | 2 00 |
| " Wm. Monroe, | Vol. 4 & 5, | 4 00 |

**MICHIGAN.**

| | | |
|---|---|---|
| Dr. S. P. Root, | Vol. 4, | $2 00 |
| " M. Mason, | " 4, | 2 00 |
| " J. T. Keables, | " 4, | 2 00 |
| " S. H. Gage, | " 4, | 2 00 |
| " O. C. Otis, | " 5, | 2 00 |
| " J. B. W. Lewis, | " 4, | 2 00 |

**MISSOURI.**

| | | |
|---|---|---|
| Dr. Wm. Curless, | Vol. 5, | $2 00 |

